# Postoperative Radiotherapy Contributes to the Survival Benefit of Breast-Conserving Therapy over Mastectomy

**DOI:** 10.1155/2022/4145872

**Published:** 2022-12-28

**Authors:** Chu-Ying Chen, Si-Yue Zheng, Gang Cai, Cheng Xu, Rong Cai, Min Li, Kun-Wei Shen, Xiao-Song Chen, Dan Ou, Wei-Xiang Qi, Lu Cao, Jia-Yi Chen

**Affiliations:** ^1^Department of Radiation Oncology, Ruijin Hospital, Shanghai Jiaotong University School of Medicine, Shanghai, China; ^2^Comprehensive Breast Health Center, Ruijin Hospital, Shanghai Jiaotong University School of Medicine, Shanghai, China

## Abstract

**Purpose:**

A survival benefit of breast-conserving therapy (BCT) over mastectomy has been shown in recent studies. This study aimed to explore differences in recurrence patterns between BCT and mastectomy and clarify the contribution of radiotherapy (RT) to the survival benefit of BCT.

**Methods:**

Consecutive patients with pT1-2/pN0-1/M0 breast cancer between 2009 and 2015 in our institution were retrospectively reviewed and compared in matched cohorts using 1 : 1 propensity score matching (PSM).

**Results:**

A total of 2370 patients were enrolled with a median follow-up of 75 (3–148) months. In the cohort without regional nodal irradiation (RNI), WBI was associated with significantly increased 10-year relapse-free survival (RFS), distant metastasis-free survival (DMFS), and regional recurrence-free survival (RRFS) compared with mastectomy alone. There were 419 pairs in the cohort without RNI and 87 pairs in the cohort with RNI after PSM. In the PSM cohort, improved 10-year RFS (95.4% vs. 82.7%, *p* < 0.05), DMFS (97.4% vs. 84.1%, *p* < 0.05), and RRFS (99.1% vs. 95.5%, *p* < 0.05) were observed in WBI compared with mastectomy alone. Regarding the first recurrence event, WBI demonstrated a significantly lower cumulative rate of distant metastases than mastectomy alone. There was no significant difference in survival outcomes between WBI plus RNI and PMRT before and after the PSM. In patients without RNI, mastectomy alone was significantly associated with unfavorable RFS (HR = 2.3, 95% CI 1.2–4.5, *p* < 0.05) and DMFS (HR = 2.5, 95% CI 1.1–5.8, *p* < 0.05).

**Conclusion:**

This study found the benefit of RFS and DMFS in BCT patients compared with those treated with mastectomy without RNI but not in those treated with RNI. We hypothesized that RT played an important role in reducing the risk of regional recurrence and distant metastases.

## 1. Introduction

Randomized trials in the 1980s demonstrated equivalent long-term survival between patients undergoing breast-conserving therapy (BCT) (a combination of breast-conserving surgery (BCS) and postoperative radiotherapy (RT)) and those receiving mastectomy [1–4]. Since then, surgical techniques, systemic therapies, and RT techniques have evolved.

Recent population-based studies of contemporary treatment have found superior survival with BCT over mastectomy in early breast cancer [5–12]. Van Maaren and colleagues found better 10-year distant metastasis-free survival (DMFS) of BCT over mastectomy-alone in T1N0 patients and most patients in this subgroup received no adjuvant systemic therapy [6]. Onitilo et al. reported a better survival of BCT over mastectomy-alone and hypothesized an association of RT with therapeutic benefits [13]. One explanation for this finding is that the application of the modern RT technique spares normal tissue [14]. Although the 15-year follow-up of the EORTC 22922 study confirmed the advantage of regional nodal irradiation (RNI) in breast cancer recurrence and mortality risk, its role was not evaluated in randomized studies comparing BCT with mastectomy [15]. Locoregional recurrence (LRR) and distant metastasis (DM), as two important surrogates proved to be reduced by 50% with RT in a recently published EBCTCG meta-analysis, were not reported in previous studies [16]. These findings imply that modern RT technique might play an increasing role in the survival benefit of BCT over mastectomy.

Currently, although BCT remains the first treatment recommendation for T1-2, N0-1 BC patients in international guidelines, its therapeutic outcomes in comparison with mastectomy and the effect of RT on survival benefit remain to be explored and this is the aim of the current study.

## 2. Methods

### 2.1. Study Population

Consecutive patients with pT1-2, pN0-1, and M0 breast cancer who were treated with BCT or mastectomy between 2009 and 2015 in Ruijin Hospital were retrospectively reviewed. BCT consisted of BCS and whole breast irradiation (WBI). Patients with bilateral breast cancer, histologically confirmed sarcoma, receiving neoadjuvant systemic therapy, without WBI after BCS or without RNI in PMRT were excluded. This study was approved by the institutional Medicine Review Board, and a waiver of consent was obtained due to its retrospective nature.

Patient-related, tumor-related, and treatment-related characteristics were collected. The tumor was staged according to the American Joint Committee on Cancer (AJCC) Cancer staging manual (7th edition). Estrogen receptor (ER) and progesterone receptor (PR) statuses were assessed using immunohistochemical (IHC) analysis and considered positive if ≥10% of cells staining positive. A positive human epidermal growth factor receptor 2 (HER2) status was defined as an expression level intensity of 3+ on IHC or a gene amplification ratio >2.2 by fluorescence in situ hybridization (FISH). ER, PR, HER2, and Ki-67 were used to classify patients into 5 molecular subtypes: luminal *A*-like, luminal *B*-like (HER2-negative), luminal *B*-like (HER2-positive), HER2-positive (nonluminal), and triple-negative breast cancer (TNBC) according to the St Gallen 2013 consensus guidelines [17].

### 2.2. Treatment

The surgical treatment options (BCS or mastectomy) were based on the tumor characteristics and patient's preference. Sentinel lymph node biopsy (SLNB) was performed in patients with clinically negative lymph nodes (LNs), and axillary lymph node dissection (ALND) was performed in patients with clinically positive LNs or positive SLNB.

The choice of adjuvant therapy was decided by a multidisciplinary team consisting of breast surgeons, medical oncologists, radiation oncologists, and others when needed as previously described [18].

The dose prescription to the whole breast/chest wall with or without regional nodes was 45–56 Gy in 25–28 fractions. A tumor bed boost (TBB) was delivered with a dose of 10–16 Gy in 5–8 fractions following WBI. The supra/infraclavicular CTVs are kept constant for RNI in patients with positive lymph nodes, internal mammary nodes and low axilla at the discretion of the radiation oncologist.

### 2.3. Statistical Analysis

Local recurrence (LR) was defined as any recurrence in the ipsilateral breast or chest wall. Regional recurrence (RR) was defined as recurrence in the ipsilateral axillary, supra/infraclavicular, or internal mammary nodes. Distant metastasis (DM) was defined as any evidence of metastatic disease beyond the locoregional region. Relapse-free survival (RFS) events included LR, RR, DM, and death from breast cancer. All statistical endpoints were calculated from the date of surgery to the defined events, including breast cancer-specific survival (BCSS), local recurrence-free survival (LRFS), regional recurrence-free survival (RRFS), distant metastasis-free survival (DMFS), and RFS.

Categorical variables were assessed using Pearson's *χ*^2^ test or Fisher's exact test as appropriate. One-to-one propensity score matching (PSM) analysis was performed to control for selection bias between the 2 cohorts: a cohort without RNI (WBI versus mastectomy-alone) and a cohort with RNI (WBI plus RNI versus PMRT). The patients were matched on age, pT/pN stage, pathology, grade, molecular subtypes, adjuvant chemotherapy, endocrine therapy, and targeted therapy. Survival curves were estimated using the Kaplan–Meier method and compared by the log-rank test. A Cox proportional hazards model was used for multivariate analysis, including predictive variables. All hazard ratios and hazard ratio confidence interval bounds for the categorical variables were rounded to a single decimal place. A two-sided *p* value <0.05 was considered statistically significant. Statistical tests were performed using SPSS version 22.0.

## 3. Results

### 3.1. Patient Characteristics

A total of 2370 patients were enrolled, of whom 520 (21.9%) received BCT and 1850 (78.1%) received mastectomy ± RT. Among patients treated with BCT, 429 (82.5%) received WBI, and 91 (17.5%) received WBI plus RNI. Among patients treated with mastectomy, 220 out of 1850 (11.9%) patients received PMRT. The patient characteristics are shown in Table 1. Compared with those treated with mastectomy, patients who received BCT were more likely to be <50 years of age and have pT1N0 or pT1N1 disease. The distribution of molecular subtypes also varied significantly between WBI and mastectomy alone. In addition, the proportion of patients with hormone receptor (HR) positive who received endocrine therapy was higher in WBI than in mastectomy.

### 3.2. Therapeutic Outcomes between BCT (WBI ± RNI) and Mastectomy (Mastectomy ± RT)

The median follow up was 75 (3–148) months. BCT patients had a significantly better 10-year RFS (94.8% vs. 83%, *p* < 0.05), RRFS (99.2% vs. 91.1%, *p* < 0.05), and DMFS (96.4% vs. 87.4%, *p* < 0.05) over mastectomy. The 10-year BCSS and LRFS rates were comparable between BCT and mastectomy (*p* > 0.05). In multivariate analysis, mastectomy was identified as an independent prognostic factor associated with poor RFS (HR = 2.8, 95% CI 1.7–4.7, *p* < 0.05), RRFS (HR = 7.3, 95% CI 2.3–23.3, *p* < 0.05), and DMFS (HR = 2.8, 95% CI 1.5–5.0, *p* < 0.05), as shown in Tables 2, S1, and S2.

### 3.3. Therapeutic Outcomes in the Cohort without RNI (WBI vs. Mastectomy Alone)

There were 429 patients with WBI and 1630 patients with mastectomy alone. Patients in the WBI group had significantly better 10-year RFS (95.5% vs. 83%, *p* < 0.05), RRFS (99.1% vs. 90.7%, *p* < 0.05), and DMFS (97.4% vs. 87.7%, *p* < 0.05) than those in the mastectomy alone group, whereas no statistically significant difference was found in BCSS or LRFS.

**Figure 1:**
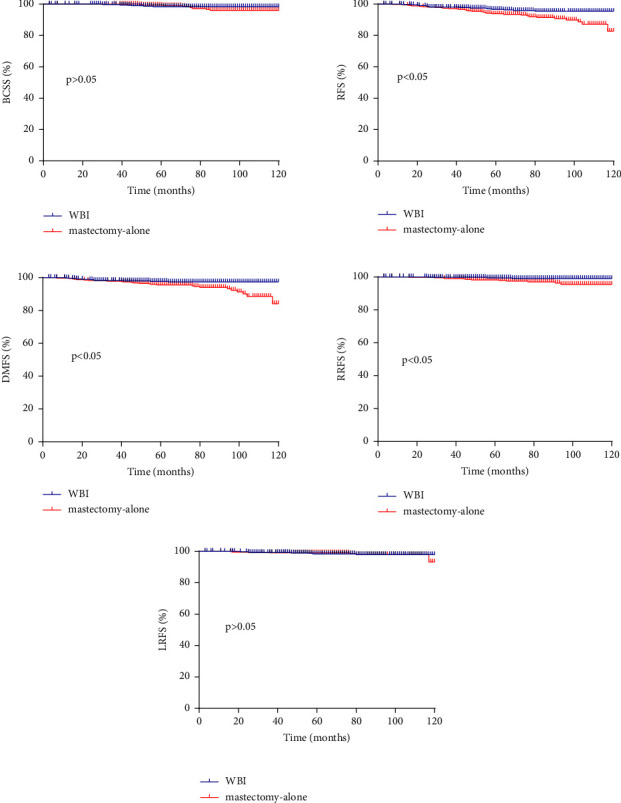
Comparisons of breast cancer-specific survival, relapse-free survival, distant metastasis-free survival, regional recurrence-free survival, and local recurrence-free survival curves between WBI and mastectomy alone after PSM.

Using PSM to balance the baseline characteristics, 419 pairs of patients were analyzed (Table 1). Patients in the WBI group consistently had a higher 10-year RFS (95.4% vs. 82.7%, *p* < 0.05), RRFS (99.1% vs. 95.5%, *p* < 0.05) and DMFS (97.4% vs. 84.1%, *p* < 0.05), while there was no significant difference in 10-year BCSS and LRFS (Figure 1). Multivariate analysis confirmed that mastectomy alone was an independent prognostic factor for worse RFS (HR = 2.3, 95% CI 1.2–4.5, *p* < 0.05) and DMFS (HR = 2.5, 95% CI 1.1–5. 8, *p* < 0.05) (Tables 2 and S1). Mastectomy alone was also unfavorable for RRFS with borderline significance (HR = 3.9, 95% CI 1.0–15.7, *p*=0.054) (Table S2). With regard to patterns of first failure, the WBI group showed a significantly lower cumulative rate of distant metastases (3.6% vs. 7.4%, *p* < 0.05) (Table 3).

### 3.4. Therapeutic Outcomes in the Cohort with RNI (WBI plus RNI vs. PMRT)

**Figure 2:**
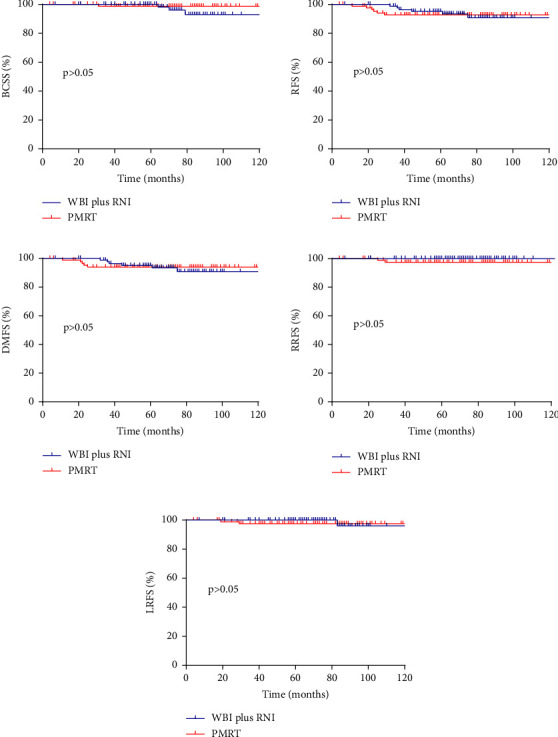
Comparisons of breast cancer-specific survival, relapse-free survival, distant metastasis-free survival, regional recurrence-free survival, and local recurrence-free survival curves between WBI plus RNI and PMRT after PSM.

In the cohort with RNI, no significant difference between WBI plus RNI and PMRT in terms of BCSS, RFS, DMFS, RRFS, and LRFS was found. After PSM, 87 pairs of patients were included (Table 1). There was no difference in BCSS, RFS, DMFS, RRFS, or LRFS rates at 10 years in the matched cohort (Figure 2). Similarly, the patterns of the first recurrence between WBI plus RNI and PMRT were comparable (Table 3).

## 4. Discussion

In the present study, we compared the survival outcomes of early-stage breast cancer patients who received BCS with WBI or mastectomy according to all parameters related to survival. We initially divided the patients into four groups according to the treatment strategy (BCS + WBI, mastectomy-alone, BCS + WBI with RNI, and mastectomy + PMRT), and our study found that improved survival was observed only in the comparison of BCT vs. mastectomy alone. Prior to the present study, several researchers attributed the survival benefit of BCT over mastectomy to postoperative radiotherapy after BCS without providing information on the field of radiation or the recurrence rates [11, 13, 19, 20]. Onitilo and colleagues made comparisons between BCS and mastectomy and found that OS was similar between BCS and mastectomy regardless of RT, but OS was better in the BCT group than in the mastectomy alone group, which was likely to be related to the addition of RT [13]. A study from the Netherlands found a survival benefit in the T1N0 subgroup in which most patients did not receive adjuvant systemic therapy after BCT or mastectomy-alone; therefore, the improvement in survival in the BCT group was attributed to RT [6]. Our study further investigated whether RT might play an essential role in the survival benefit from risk reduction of RR and DM in BCT. Kaplan–Meier survival analysis demonstrated that there was a significantly improved 10-year RFS, DMFS, and RRFS among the BCS with WBI group when compared to mastectomy alone before and after PSM. This was further confirmed by multivariate analysis, which showed that mastectomy alone was associated with worse DMFS, RRFS, and RFS in the matched cohort. However, no survival difference was observed between WBI plus RNI and PMRT among patients with postoperative RT, indicating that the surgery itself possibly had no influence on survival. It could be hypothesized that RT contributed greatly to the risk reduction of RR and DM, resulting in the benefit of RRFS and DMFS in BCT over mastectomy.

Our results are inconsistent with previous outcomes of randomized trials conducted in the 1980s showing equivalent survival between BCT and mastectomy [1–4]. The inconsistency may be explained by the fact that these trials were conducted before the modern era, and screening programs, surgical techniques, systemic therapies, and RT techniques have developed dramatically. A pooled analysis found that mastectomy significantly reduced the risk of locoregional recurrence when compared with BCT in previous trials [21], but the rate of local recurrence after BCT has fallen over time [22]. In addition, all of the BCT patients in our analysis received TBB after whole breast irradiation, which would reduce the risk of local recurrence [23], while the BCT patients only received postoperative radiotherapy without TBB in the NSABP B-06 trial [2].

The trend toward a decreased risk of regional recurrence associated with RT between WBI and mastectomy alone is probably due to the lower incidence of axillary recurrence as first recurrence, which was halved in the WBI group compared with mastectomy alone (0.7% vs. 1.4%) in the PSM cohort. This is in line with other studies providing evidence of decreased axillary recurrence in BCS plus RT compared with mastectomy without RT, even in low-risk patients [19, 20]. In addition, the results from the ACOSOG Z0011 and IBCSG-23-01 indicated that the omission of ALND did not compromise 10-year OS in 1-2 SLNB-positive or SLNB micrometastasis patients [24, 25], which reflected potential protection against axillary recurrence by incidental irradiation originating from WBI. It was estimated that more than 50% of axillary level I and 20%–30% of level II receives 95% of the prescribed radiation dose using tangential fields [26].

Given that subclinical radiation dose to the axilla contributes significantly to the regional control, a decreased risk of distant metastasis in BCT with WBI patients over mastectomy alone probably accounts for the major part of the survival benefit, as our results showed better DMFS of WBI over mastectomy only, which had been found in previous studies [20, 27, 28]. In both the MA.20 and EORTC 22922 studies, a decreased rate of DM with the addition of RNI was found [29, 30]. This was possibly attributed to a direct link between the decreased risk of regional recurrence and possible subsequent distant metastasis. The results from the EBCTCG meta-analysis showed that RT after BCS reduces the risk of recurrence and death from breast cancer, indicating that RT plays an important role in killing microscopic tumor foci after surgery and therefore reduces the potential for both local recurrence and distant metastasis [16]. Overgaard et al. found that the 10-year disease-free survival was 48% among women with PMRT and 34% among those without PMRT in high-risk patients (*p* < 0.001) and a similar magnitude of benefit in 10-year OS from 45% without PMRT to 54% with PMRT (*p* < 0.001), showing that the addition of RT reduces locoregional recurrences and subsequent distant metastasis that improves survival [31]. However, although we found better DMFS in WBI compared with mastectomy alone, no improvement in BCSS was found. The fact that this did not translate into better BCSS might be explained by the salvage treatment after distant metastases and the 75 months of median follow-up time, which was not long enough to detect differences in the specific survival.

T1-2 breast cancer patients with negative LNs account for a large proportion of breast conservation. As RT is not routinely recommended in this group of patients when undergoing mastectomy, patients receiving BCS are more likely to be provided the risk reduction of RR and DM with subsequent RT. Improved survival associated with BCT compared with mastectomy alone was also found in the early stage, node-negative breast cancer in another study [10]. For patients with 1–3 positive LNs, expert consensus on the role of PMRT remains controversial [32]. A meta-analysis from EBCTCG found that PMRT reduced locoregional recurrence and mortality rates in this group [33], whereas some retrospective analyses observed no significant differences in BCSS or overall survival (OS) with the addition of PMRT in the era of modern systemic therapy [34, 35]. Due to the debate on this issue, a small portion of patients with pN1 undergoing mastectomy did not receive PMRT in our study and might have received RT if BCS had been used.

Although other studies have also shown improved survival with BCT compared with mastectomy in node-positive patients [36, 37], our study is the first to separately compare BCS + WBI-only vs. mastectomy without PMRT and BCS + WBI with RNI vs. mastectomy with PMRT including RNI. By comparing these two cohorts with PSM analysis and multivariate analysis, in early-stage patients, mostly T1-2N0, BCS + WBI showed a protective therapeutic effect in reducing the risk of regional recurrence and distant metastasis, which should be attributed to the RT. In high-risk patients who received comprehensive local-regional RT, regardless of the pattern of primary breast surgery, no significant differences in terms of local-regional recurrence or distant metastasis were found. One can hypothesize that BCS with WBI is associated with better survival than mastectomy alone; therefore, BCS is strongly recommended in early breast cancer if indicated.

As a retrospective study, selection bias was inevitable. PSM analysis balanced the major prognostic parameters, but some variables remained unbalanced. For example, the role of the oncotype DX recurrence score (RS) was not assessed in our study, although its role in guiding BCS is still uncertain [38]. The median follow-up in our study was 75 months and longer follow-up is necessary. Finally, whether the role of RT in survival varies in different molecular subtypes, as previously reported, was not explored in our study [37, 39–41].

## 5. Conclusions

Our study confirms that in early-stage breast cancer not indicated for comprehensive RNI, BCS with WBI is associated with a better prognosis than mastectomy without PMRT in terms of RFS, DMFS, and RRFS. For high-risk patients with comprehensive local-regional RT, similar survival outcomes are observed regardless of the type of breast surgery. Our results, along with the previous studies finding a survival advantage with BCT, provide evidence that this effect should be attributed to RT since it reduces the risk of regional recurrence and distant metastases rather than to the type of surgery itself. Our findings support BCT as a therapeutic choice of priority in early breast cancer.

## Figures and Tables

**Table 1:** Baseline characteristics.

	Whole cohort						Matched cohort					
	WBI	Mastectomy alone	*p*value	WBI + RNI	PMRT	*p*value	WBI	Mastectomy alone	*p*value	WBI + RNI	PMRT	*p*value
	*N* = 429	*N* = 1630		*N* = 91	*N* = 220		*N* = 419	*N* = 419		*N* = 87	*N* = 87	
Age (years)												
Median (range)	52 (27–78)	57 (23–92)		50 (31–78)	53.5 (31–78)		52 (27–78)	52 (28–92)		50 (31–78)	52 (31–78)	
<50	178 (41.5%)	477 (29.3%)	<0.05	44 (48.4%)	80 (36.4%)	<0.05	170 (40.6%)	177 (42.2%)	>0.05	40 (46.0%)	34 (39.1%)	>0.05
≥50	251 (58.5%)	1153 (70.7%)		47 (51.6%)	140 (63.6%)		249 (59.4%)	242 (57.8%)		47 (54.0%)	53 (60.9%)	

Pathological stage			<0.05			<0.05			>0.05			>0.05
T1N0	326 (76.0%)	876 (53.7%)		0 (0.0%)	0 (0.0%)		316 (75.4%)	312 (74.5%)		0 (0.0%)	0 (0.0%)	
T1N1	18 (4.2%)	114 (7.0%)		64 (70.3%)	98 (44.5%)		18 (4.3%)	14 (3.3%)		60 (69.0%)	57 (65.5%)	
T2N0	76 (17.7%)	520 (31.9%)		2 (2.2%)	7 (3.2%)		76 (18.1%)	79 (18.9%)		2 (2.3%)	4 (4.6%)	
T2N1	9 (2.1%)	120 (7.4%)		25 (27.5%)	115 (52.3%)		9 (2.1%)	14 (3.3%)		25 (28.7%)	26 (29.9%)	

Grade			<0.05			>0.05			>0.05			>0.05
I	36 (8.4%)	85 (5.2%)		4 (4.4%)	4 (1.8%)		34 (8.1%)	34 (8.1%)		3 (3.4%)	2 (2.3%)	
II	178 (41.5%)	753 (46.2%)		38 (41.8%)	99 (45.0%)		173 (41.3%)	165 (39.4%)		38 (43.7%)	30 (34.5%)	
III	155 (36.1%)	539 (33.1%)		34 (37.4%)	86 (39.1%)		153 (36.5%)	159 (37.9%)		34 (39.1%)	38 (43.7%)	
Unknown	60 (14.0%)	253 (15.5%)		15 (16.5%)	31 (14.1%)		59 (14.1%)	61 (14.6%)		12 (13.8%)	17 (19.5%)	

Histological subtype			>0.05			>0.05			>0.05			>0.05
Ductal	379 (88.3%)	1408 (86.4%)		83 (91.2%)	203 (92.3%)		369 (88.1%)	365 (87.1%)		81 (93.1%)	80 (92.0%)	
Lobular	14 (3.3%)	54 (3.3%)		1 (1.1%)	7 (3.2%)		14 (3.3%)	12 (2.9%)		1 (1.1%)	0 (0.0%)	
Other	36 (8.4%)	168 (10.3%)		7 (7.7%)	10 (4.5%)		36 (8.6%)	42 (10.0%)		5 (5.7%)	7 (8.0%)	

Molecular subtype			<0.05			>0.05			>0.05			>0.05
Luminal A-like	163 (38.0%)	593 (36.4%)		28 (30.8%)	50 (22.7%)		161 (38.4%)	158 (37.7%)		26 (29.9%)	22 (25.3%)	
Luminal B-like (HER2 negative)	110 (25.6%)	406 (24.9%)		26 (28.6%)	59 (26.8%)		109 (26%)	121 (28.9%)		26 (29.9%)	25 (28.7%)	
Luminal B-like (HER2 positive)	29 (6.8%)	180 (11.0%)		6 (6.6%)	26 (11.8%)		29 (6.9%)	30 (7.2%)		6 (6.9%)	4 (4.6%)	
HER2 positive (nonluminal)	25 (5.8%)	191 (11.7%)		9 (9.9%)	34 (15.5%)		25 (6.0%)	19 (4.5%)		9 (10.3%)	13 (14.9%)	
TNBC	84 (19.6%)	231 (14.2%)		18 (19.8%)	37 (16.8%)		84 (20.0%)	77 (18.4%)		16 (18.4%)	18 (20.7%)	
Unknown	18 (4.2%)	29 (1.8%)		4 (4.4%)	14 (6.4%)		11 (2.6%)	14 (3.3%)		4 (4.6%)	5 (5.7%)	

Adjuvant chemotherapy			>0.05			>0.05			>0.05			>0.05
No	165 (38.5%)	644 (39.5%)		3 (3.3%)	6 (2.7%)		159 (37.9%)	171 (40.8%)		3 (4.3%)	3 (3.4%)	
Yes	264 (61.5%)	969 (59.4%)		86 (94.5%)	208 (94.5%)		260 (62.1%)	247 (58.9%)		82 (94.3%)	81 (93.1%)	
Unknown	0 (0.0%)	17 (1.0%)		2 (2.2%)	6 (2.7%)		0 (0.0%)	1 (0.2%)		2 (2.3%)	3 (3.4%)	

Targeted therapy in HER2 positive tumor			>0.05			>0.05			>0.05			>0.05
No	22 (40.7%)	129 (34.5%)		3 (20.0%)	20 (33.3%)		22 (40.7%)	22 (44.0%)		3 (20.0%)	2 (11.8%)	
Yes	31 (57.4%)	234 (62.6%)		12 (80.0%)	39 (65.0%)		31 (57.4%)	26 (52.0%)		12 (80.0%)	14 (82.4%)	
Unknown	1 (1.9%)	11 (2.9%)		0 (0.0%)	1 (1.7%)		1 (1.9%)	2 (4.0%)		0 (0.0%)	1 (5.9%)	

Endocrine therapy in HR-positive tumor			<0.05			>0.05			>0.05			>0.05
No	5 (1.6%)	110 (9.1%)		1 (1.6%)	2 (1.4%)		5 (1.6%)	9 (2.8%)		1 (1.7%)	0 (0.0%)	
Yes	308 (96.9%)	1089 (90.4%)		60 (96.8%)	131 (88.5%)		301 (97.7%)	312 (97.2%)		58 (96.7%)	51 (92.7%)	
Unknown	5 (1.6%)	5 (0.4%)		1 (1.6%)	15 (10.1%)		2 (0.6%)	0 (0.0%)		1 (1.7%)	4 (7.3%)	

WBI, whole breast irradiation; RNI, regional nodal irradiation; PMRT, postmastectomy radiotherapy; TNBC, triple-negative breast cancer; HR, hormone receptor.

**Table 2:** . The multivariate analysis of risk factors for RFS in the whole cohort and in patients without RNI of the matched cohort.

Variables	Whole cohort			Patients without RNI of the matched cohort		
	HR	95% CI	*p* value	HR	95% CI	*p* value
Age (years)	1.002	0.989–1.016	>0.05	1.008	0.981–1.036	>0.05
Grade						
I	1.0			1.0		
II	3.1	0.7–12.7	>0.05	—	—	>0.05
III	4.9	1.2–20.6	<0.05	—	—	>0.05
Molecular subtype						
Luminal A-like	1.0			1.0		
Luminal B-like (HER2-negative)	1.7	1.1–2.7	<0.05	1.3	0.5–3.2	>0.05
Luminal B-like (HER2-positive)	0.9	0.4–1.7	>0.05	0.6	0.1–3.1	>0.05
HER2-positive (nonluminal)	0.9	0.4–2.0	>0.05	0.4	0.0–3.7	>0.05
TNBC	1.6	0.8–3.6	>0.05	0.6	0.1–3.5	>0.05
Adjuvant chemotherapy						
No	1.0			1.0		
Yes	1.1	0.7–1.7	>0.05	1.4	0.6–3.2	>0.05
Endocrine therapy						
No	1.0			1.0		
Yes	0.8	0.4–1.5	>0.05	0.5	0.1–2.1	>0.05
Treatment						
BCT	1.0			1.0		
Mastectomy	2.8	1.7–4.7	<0.05	2.3	1.2–4.5	<0.05

TNBC, triple-negative breast cancer; BCT, breast-conserving therapy.

**Table 3:** Patterns of the first recurrence in matched cohorts.

First recurrence	WBI		Mastectomy alone		WBI + RNI		PMRT	
	*N* = 419	%	*N* = 419	%	*N* = 87	%	*N* = 87	%
LR	8	1.9	6	1.4	0	0	1	1.1
RR	5	1.2	10	2.4	0	0	2	2.3
Axillary	3	0.7	6	1.4	0	0	0	0
Supraclavicular	2	0.5	3	0.7	0	0	2	2.3
Internal mammary	0	0	1	0.2	0	0	0	0
CBC	7	1.7	10	2.4	0	0	0	0
DM	15	3.6	31	7.4	9	10.3	7	8
Bone	4	1.0	8	1.9	5	5.7	4	4.6
Liver/lung/brain	7	1.7	16	3.8	4	4.6	1	1.1
Distant nodes	3	0.7	5	1.2	0	0	2	2.3
Other sites	1	0.2	2	0.5	0	0	0	0

LR, local recurrence; RR, regional recurrence; CBC, contralateral breast cancer; DM, distant metastasis.

## Data Availability

The data are not available due to ethical restrictions.

## Supplementary Materials

Supplementary Materials Tables S1: multivariate analysis of risk factors for DMFS in the whole cohort and in patients without RNI of the matched cohort. Tables S2: multivariate analysis of risk factors for RRFS in the whole cohort and in patients without RNI of the matched cohort.
