# Supplementary material for: Postoperative Radiotherapy Contributes to the Survival Benefit of Breast-Conserving Therapy over Mastectomy
Source: J Oncol. 2022 Dec 28;2022:4145872. doi: 10.1155/2022/4145872 (PMC9812601; doi:10.1155/2022/4145872)
Supplement: Supplementary Materials — Tables S1: multivariate analysis of risk factors for DMFS in the whole cohort and in patients without RNI of the matched cohort. Tables S2: multivariate analysis of risk factors for RRFS in the whole cohort and in patients without RNI of the matched cohort. [file 4145872.f1.docx]

TABLE S1 Multivariate analysis of risk factors for DMFS in whole cohort and in patients without RNI of the matched cohort

| Variables | whole cohort | | |  | patients without RNI of the matched cohort | | |
| --- | --- | --- | --- | --- | --- | --- | --- |
|  | HR | 95%CI | *p* value |  | HR | 95%CI | *p* value |
| Age (years) | 1.01 | 0.994-1.026 | >0.05 |  | 1.036 | 1.001-1.072 | <0.05 |
| Grade |  |  |  |  |  |  |  |
| Ⅰ | 1.0 |  |  |  | 1.0 |  |  |
| Ⅱ | 1.9 | 0.5-8.0 | >0.05 |  | - | - | >0.05 |
| Ⅲ | 3.6 | 0.8-15.6 | >0.05 |  | - | - | >0.05 |
| Molecular subtype |  |  |  |  |  |  |  |
| Luminal A-like | 1.0 |  |  |  | 1.0 |  |  |
| Luminal B-like (HER2-negative) | 1.8 | 1.1-3.1 | <0.05 |  | 1.1 | 0.4-3.6 | >0.05 |
| Luminal B-like (HER2-positive) | 1.1 | 0.5-2.4 | >0.05 |  | 0.5 | 0.1-4.2 | >0.05 |
| HER2-positive (non-luminal) | 1.0 | 0.3-3.1 | >0.05 |  | 0.6 | 0.0-8.7 | >0.05 |
| TNBC | 2.2 | 0.8-5.9 | >0.05 |  | 0.8 | 0.1-8.8 | >0.05 |
| Adjuvant chemotherapy |  |  |  |  |  |  |  |
| No | 1.0 |  |  |  | 1.0 |  |  |
| Yes | 1.2 | 0.7-1.9 | >0.05 |  | 1.8 | 0.6-5.4 | >0.05 |
| Endocrine therapy |  |  |  |  |  |  |  |
| No | 1.0 |  |  |  | 1.0 |  |  |
| Yes | 1.2 | 0.5-2.7 | >0.05 |  | 0.7 | 0.1-5.6 | >0.05 |
| Treatment |  |  |  |  |  |  |  |
| BCT | 1.0 |  |  |  | 1.0 |  |  |
| Mastectomy | 2.8 | 1.5-5.0 | <0.05 |  | 2.5 | 1.1-5.8 | <0.05 |

*TNBC* triple-negative breast cancer; *BCT* breast conserving therapy

TABLE S2 Multivariate analysis of risk factors for RRFS in whole cohort and in patients without RNI of the matched cohort

| Variables | whole cohort | | |  | patients without RNI of the matched cohort | | |
| --- | --- | --- | --- | --- | --- | --- | --- |
|  | HR | 95%CI | *p* value |  | HR | 95%CI | *p* value |
| Age (years) | 0.992 | 0.972-1.013 | >0.05 |  | 0.952 | 0.900-1.006 | >0.05 |
| Grade |  |  |  |  |  |  |  |
| Ⅰ | 1.0 |  |  |  | 1.0 |  |  |
| Ⅱ | 1.4 | 0.3-6.0 | >0.05 |  | - | - | >0.05 |
| Ⅲ | 1.5 | 0.3-6.9 | >0.05 |  | - | - | >0.05 |
| Molecular subtype |  |  |  |  |  |  |  |
| Luminal A-like | 1.0 |  |  |  | 1.0 |  |  |
| Luminal B-like (HER2-negative) | 2.4 | 1.2-5.0 | <0.05 |  | 0.7 | 0.2-3.5 | >0.05 |
| Luminal B-like (HER2-positive) | 0.7 | 0.2-2.4 | >0.05 |  | - | - | >0.05 |
| HER2-positive (non-luminal) | 1.7 | 0.4-6.3 | >0.05 |  | 0.9 | - | >0.05 |
| TNBC | 3.5 | 1.0-11.9 | 0.05 |  | - | - | >0.05 |
| Adjuvant chemotherapy |  |  |  |  |  |  |  |
| No | 1.0 |  |  |  | 1.0 |  |  |
| Yes | 0.7 | 0.4-1.2 | >0.05 |  | 0.8 | 0.2-3.6 | >0.05 |
| Endocrine therapy |  |  |  |  |  |  |  |
| No | 1.0 |  |  |  | 1.0 |  |  |
| Yes | 0.8 | 0.3-2.2 | >0.05 |  | - | - | >0.05 |
| Treatment |  |  |  |  |  |  |  |
| BCT | 1.0 |  |  |  | 1.0 |  |  |
| Mastectomy | 7.3 | 2.3-23.3 | <0.05 |  | 3.9 | 1.0-15.7 | >0.05 |

*TNBC* triple-negative breast cancer; *BCT* breast conserving therapy
